# Multi-Omics-Based Discovery of Plant Signaling Molecules

**DOI:** 10.3390/metabo12010076

**Published:** 2022-01-13

**Authors:** Fei Luo, Zongjun Yu, Qian Zhou, Ancheng Huang

**Affiliations:** Key Laboratory of Molecular Design for Plant Cell Factory of Guangdong Higher Education Institutes, SUSTech-PKU Institute of Plant and Food Science, Department of Biology, School of Life Sciences, Southern University of Science and Technology, Shenzhen 518055, China; 12031117@mail.sustech.edu.cn (F.L.); 11930151@mail.sustech.edu.cn (Z.Y.)

**Keywords:** plant signaling molecules, multi-omics, secondary metabolites, structures and functions

## Abstract

Plants produce numerous structurally and functionally diverse signaling metabolites, yet only relatively small fractions of which have been discovered. Multi-omics has greatly expedited the discovery as evidenced by increasing recent works reporting new plant signaling molecules and relevant functions via integrated multi-omics techniques. The effective application of multi-omics tools is the key to uncovering unknown plant signaling molecules. This review covers the features of multi-omics in the context of plant signaling metabolite discovery, highlighting how multi-omics addresses relevant aspects of the challenges as follows: (a) unknown functions of known metabolites; (b) unknown metabolites with known functions; (c) unknown metabolites and unknown functions. Based on the problem-oriented overview of the theoretical and application aspects of multi-omics, current limitations and future development of multi-omics in discovering plant signaling metabolites are also discussed.

## 1. Introduction

Small molecules produced by plants play vastly diverse roles in nature, amongst which signaling and communication are two of the most important aspects. Plant metabolites are broadly classified into primary and secondary metabolites. Primary metabolites are ubiquitous to all plants whereas secondary metabolites are specifically produced by certain plants, tissues and cells and in most cases elicited under certain conditions. It is estimated that there are over one million metabolites produced throughout the plant kingdom [1]. Secondary metabolites (including but not limited to terpenes, phenylpropanoids and alkaloids) are important signaling molecules that convey information in a spatial–temporal-specific manner [2]. We define signaling molecules as those small plant metabolites that can be perceived by living organisms and trigger or participate in signal transduction.

These metabolites can serve as signaling molecules during plant growth and development, initiating and coordinating plant developmental programs. In the meantime, they can “liaise” with external environments and other living organisms, fulfilling the subtle demands for plant health and growth. Plant hormones including jasmonic acid [3], abscisic acid [4], brassinosteroids [5,6], auxin [7], gibberellins [8], strigolactones [9], ethylene [10] and salicylic acids [11] are well known signaling molecules that participate in numerous aspects of plant growth, defense and plant–environment interactions. These compounds are essential for plant growth and development, yet their specific roles and the ways they function can vary drastically among different plant species and under specific environmental conditions. Secondary metabolites were well-known for their direct impacts on herbivores and pathogens in plant defense. More recently, their functions as signaling molecules that indirectly aid plants in overcoming stresses are gradually being unveiled. For instance, triterpenes including the oat antifungal avenacin precursor β-amyrin [12] and thalianol-derived triterpenes from *Arabidopsis thaliana* [13] were found to participate in plant root growth and development, with β-amyrin affecting the oat root epidermal cell patterning and thalianol derivatives impacting *A. thaliana* root length, respectively. Defense compound glucosinolate can also influence plant growth via its degradation product indole-3-carbinol, which inhibits root elongation by competing directly with auxin as a signaling molecule, so as to maintain the balance between plant growth and plant defense [14]. Other plant metabolites such as flavones apigenin and luteolin were recently found to be able to promote maize growth and nitrogen acquisition via recruiting beneficial bacteria of the taxa *Oxalobacteraceae* [15]. Such indirect effects of secondary metabolites on plant performance were also observed in maize. Maize roots exuded well-known defense compounds, benzoxazinoids, that altered the root-associated microbiota in soils, which, in turn, exerted a prolonged impact on the growth and herbivore resistance of maize in the next generation [16].

**Figure 1:**
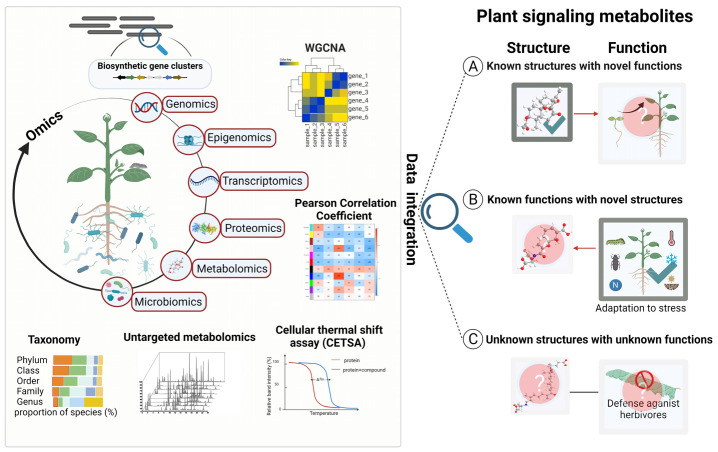
Overview of multi-omics for plant signaling metabolite discovery. Key tools at the different levels of omics for plant signaling discovery are highlighted on the left panel. The different categories (**A**–**C**) of plant signaling metabolites awaiting discovery are depicted on the right panel. WGCNA, weighted gene co-expression network analysis.

The aforementioned examples demonstrate that even some of the best-known plant metabolites still have unknown functions awaiting discovery. Moreover, the majority of plant metabolites discovered so far have only been chemically/structurally characterized and not yet been assigned a definite function in nature. The plant metabolites that we have already discovered might actually represent only the tip of the iceberg regarding the metabolic diversity of plants, as implicated by the numerous uncharacterized predicted biosynthetic genes present in plant genomes [17]. Current research concerning the discovery of plant signaling metabolites can be broadly classified into three categories: (a) plant metabolites with known structures but unclear functions; (b) plant metabolites with unknown structures but implicated functions; (c) plant metabolites with yet to be determined structures and functions (Figure 1). The difficulties in discovering plant signaling metabolites under these three scenarios also vary.

There are a few major challenges impeding the discovery of plant signaling molecules: (i) the content of plant signaling metabolites are usually very low; (ii) plant signaling metabolites are often under dynamic metabolism (i.e., they are actively being synthesized as well as being catabolized and secreted); (iii) plant signaling metabolites normally have characteristic spatial–temporal distributions (they can respond to the upstream signal transduction cascade, including those from the environment, growth and developmental programs at specific stages); (iv) they have extremely diverse physical and chemical properties that demand customized analytical and assay methods; (v) they have diverse specialized functions that can only be captured under specific spatial and temporal conditions.

Addressing these challenges requires interdisciplinary approaches. Multi-omics is a powerful and indispensable integrated technique that has greatly accelerated the discovery of plant signaling metabolites via systematic comparative analysis of large datasets (Figure 1). Experimental designs and technical application are critical for the successful implementation of multi-omics for discovering plant signaling metabolites. This review synthesizes the technical features and limitations of multi-omics and discusses effective strategies for implementation with recent successful examples in discovering plant signaling metabolites for the purpose of providing guidance for the effective application of multi-omics technologies in uncovering the structures and functions of plant metabolites.

## 2. Multi-Omics as a Powerful Tool for Uncovering Plant Signaling Metabolites

Biological networks are highly complex, interconnected and tightly regulated. Plant metabolites are the output of the Central Dogma, closely related to phenotypes and associated with various aspects of cellular processes ranging from biosynthesis and catabolism to regulation, transport, mode of action and their interactions with environmental changes. Each of the related aspects provides an entry point for investigating plant signaling metabolites. These entry points correspond well with the different levels of omics (including genomics, epigenomics, transcriptomics, proteomics, metabolomics and microbiomics) that are currently available. Different levels of omics techniques will have to be employed in a combinatorial fashion to reveal a relatively complete picture of plant signaling metabolites. Depending on the nature of the study, and the current knowledge of the structures and functions of the metabolites of interest, one might design experiments with specific focus on one or two omics techniques. Nevertheless, an in-depth grasp of the technical features and research aims are critical for the successful execution of multi-omics.

### 2.1. Features of Multi-Omics, Including Genomics, Epigenomics, Transcriptomics, Proteomics, Metabolomics and Microbiomics

Multi-omics refers to the integrated application of more than one type of large dataset analysis, including genomics, epigenomics, transcriptomics, proteomics, metabolomics and microbiomics. To better understand biological activities at a system level, traditional single-omics research is rarely comprehensive enough and requires the integrated multi-omics data for global analysis of biological systems [18], and multidimensional analysis as well as multi-stage development analysis are increasingly used to understand biological mechanisms and deepen our understanding of plants and environment. Each single omics has its own feature that could compensate for limitations of the other omics techniques.

#### 2.1.1. Genomics—The Source Code for Discovering Plant Signaling Metabolites

Genomics involves the study of complete DNA sets in organisms, including all its genes, their sequences, arrangements and architecture, providing perspective for looking into biological problems from the most basic code of life DNA. DNA carries instructions for transcription (promoters, untranslated regulatory regions and splicing sites), translation (start and stop codons) and specific functions of a gene (coding sequence) [19]. Genomics features underlying transcriptional and translational regulation, biosynthesis and the transport of plant metabolites can be utilized for systematic mining at single or multiple genome scales for discovering plant signaling metabolites. Driven by advances in high-throughput DNA sequencing technologies such as Illumina HiSeq, PacBio and Nanopore sequencing, more than 600 plant genomes have been sequenced and made publicly available [20]. The majority of genomes deposited have been structurally and functionally annotated, thus, can be exploited for mining biosynthetic genes and other genomic features concerning plant metabolites. Protein family domains and physical arrangements of the corresponding genes (e.g., whether or not colocalized) in the genomes can be used for predicting the types of enzymes and potential metabolic products derived thereof [21]. For instance, a rare type of terpene compound, namely sesterterpenes, which are convergently synthesized by plants and fungi, were discovered via investigating the metabolic output of an interesting colocalization phenomenon of genes containing the prenyltransferase (Polyprenyl_synt (PT)) and terpene synthase (Terpene_synth C (TPS)) domains in plant genomes [22,23]. Gene-guided approaches have also been employed to discover the precursor gene encoding peptide with the BURP domain (Pfam 03181) and core ribosomal peptide(s) for bioactive compound lyciumin biosynthesis. The newly discovered genomic features underpinning lyciumin biosynthesis enabled the customized tblastn search in plant genomes for genes encoding BURP domain proteins to identify ribosomally synthesized candidates and post-translationally modified peptides (RiPPs) in *Amaranthaceae*, *Fabaceae*, *Rosaceae* and *Solanaceae* families [24]. With the usage of plant genomic sequence, protein annotation and gene expression profile, a few bioinformatic tools including plantiSMASH [21], phytocluster [25] and clusterfinder [26,27] have been developed to predict plant biosynthetic gene clusters (BGCs) from plant genomes, which will certainly facilitate the discovery of plant signaling metabolites.

#### 2.1.2. Epigenomics—The Gatekeeper for Plant Metabolite Biosynthesis

DNA in cells is wrapped around histone proteins H1, H2A, H2B, H3 and H4, which form spool-like structures that enable very long DNA molecules to be wrapped up neatly into chromosomes inside the cell nucleus. DNA and histones can undergo reversible chemical modifications like DNA methylation or histone methylation, acetylation, phosphorylation and adenylation, the complete set of which in cells are heritable without changing the DNA sequence, termed epigenome. Epigenomics unitizes high-throughput technologies to decipher epigenome landscapes based on comprehensive analyses.

Epigenome landscapes are tightly associated with gene activity and expression, controlling the production of proteins and metabolites under a specific condition via altering chromatin conformation or transcription regulator recruitment. DNA methylation is one well-known epigenomic process with methyl groups being added to the bases of a DNA molecule at specific sites, switching the genes on or off by altering interactions between the DNA and methyl group reading proteins. Epigenomics technologies including chromatin immunoprecipitation–sequencing (ChIP–seq) and Assay for Transposase-Accessible Chromatin using sequencing (ATAC–Seq) [28] enable detection of global chemical modifications associated with various aspects of plant signaling metabolites, thereby providing another perspective for looking into metabolite biosynthesis and regulation. Via ChIP–seq analysis, plant triterpenes thalianol and marneral biosynthetic gene clusters were found to be regulated by histone modification with histone 3 lysine trimethylation (H3K27me3) and the histone2 variant H2A.Z reported to repress and activate the thalianol and marneral gene clusters, respectively [29]. Besides triterpenes, camalexin biosynthesis genes were also found to contain epigenetic marks with H3K18ac and H3K27me3 found to activate and repress gene expression, respectively [30]. Similarly, the diterpene gene cluster responsible for the biosynthesis of the antifungal diterpene, ent-5,10-diketo-casbene, was recently found to also be under the regulation of epigenetic modifications with H3K27me3 acting as a repression mark [31]. Epigenomics can yield meaningful information for discovering the regulatory mechanism of plant signaling metabolite biosynthesis, especially when used together with other omics techniques.

Apart from plants having complete genome sequences as mentioned above, epigenomics may also be applied to decouple the regulatory mechanisms underlying plant signaling metabolite biosynthesis in non-model plants that lack a whole genome, using techniques such as epiGBS, reference-free reduced representation bisulfite sequencing [32] for exploration and comparative analysis of DNA methylation de novo. This method could help to profile epigenetic regulation patterns and understand how epigenetic regulatory mechanisms affect metabolite biosynthesis in non-model plants.

#### 2.1.3. Transcriptomics—Snapshots of Gene Expression under Specific Spatial–Temporal Conditions

Transcriptomics is used to study all types of RNA transcripts including messenger RNAs (mRNAs), microRNAs (miRNAs) and long noncoding RNAs (lncRNAs) present in a sample under specific conditions. As one of the most widely used high-throughput sequencing methods, modern transcriptomics technology has developed from bulk RNA sequencing (RNA-seq) at the tissue or population level to single-cell RNA-seq at the individual cell level using nanopore sequencing and 10× Genomics single-cell sequencing [33]. In contrast to the high cost of plant genome sequencing, RNA-seq is a cost-efficient and facile approach to obtain snapshots of gene expression at a cell/organ/tissue under the conditions being studied. Transcriptomics data can reveal information related to many aspects of RNAs, including expression levels, functions, locations, trafficking, degradation, structures of transcripts and their parent genes, with regard to start sites, 5′ and 3′ untranslated regions (UTR), splicing patterns, alternative polyadenylation profiles and post-transcriptional modifications [34].

Transcriptomics is particularly useful when no genome information is available for the plant to be studied, as RNA-seq data can be assembled de novo to retrieve coding sequences (CDS) of biosynthetic genes. This has proven to be a powerful tool for discovering biosynthetic genes responsible for the synthesis of metabolites (e.g., colchicine alkaloid [35] and protolimonoid biosynthesis [36]) in medicinal plants. Furthermore, signaling metabolite-associated genes display similar expression patterns for certain biological events [37]. RNA-seq is extremely powerful for uncovering patterns of genes relevant to biological (e.g., developmental and environmental) events, exposing links between metabolite biosynthesis and genes related to their functions, hence, facilitating the discovery of metabolite structures and functions. By using the gene expression matrix from RNA-seq data analysis, various co-expression analysis approaches, including weighted gene co-expression network analysis (WGCNA) [38], hierarchical clustering [39], Pearson Correlation Coefficient (PCC) [40], Highest Reciprocal Rank (HRR) [41], Mutual Rank (MR) [42] and Self-Organizing Map (SOM) [43], have been successfully applied in identifying candidate genes involved in plant-specialized metabolic pathways by utilizing known biosynthetic genes as a bait [44]. For instance, based on a SOM analysis of oat (Avena species) transcriptomic data for six tissues, six transcripts of the known antifungal avenacin biosynthetic pathway genes clustered to a node of the self-organizing map, indicating the co-expression of these genes. Within the transcripts clustered with the avenacin biosynthetic pathway genes in 100% of self-organizing map runs, nine transcripts were identified as candidate avenacin glycosyltransferase genes (UGT). Combing the phylogenetic analysis of the predicted amino acid sequences of the nine new candidate UGTs with Agrobacterium-mediated transient expression assay, AsTG1 and AsUGT91G16 were proven to form part of the avenacin biosynthetic gene cluster [45]. 

#### 2.1.4. Proteomics—The Yet to Flourish Tool for Plant Signaling Metabolite Discovery

Proteins translated from mRNA are effectors of biological functions, catalyzing reactions, transmitting signals and creating cellular support structures. Proteomics studies the complete set of protein abundance, structures, functions, post-translational modifications and protein–protein/metabolite interactions in a living organism under given conditions. Protein abundance is closely related to transcript abundance but more dynamic due to miscellaneous degradation and modification mechanisms present in plant cells. Some biosynthetic enzymes responsible for the synthesis of secondary metabolites are actually regulated by post-translational modifications [46]. Proteomics can also be used to improve the functional annotation of genes in plant genomes, reducing difficulties for future bioinformatics analysis and cloning efforts [47]. Moreover, some properties of proteins (e.g., solubility/melting points) can change systematically when interacting with proteins or metabolites, providing opportunities to probe protein–protein and protein–metabolite interactions using methods like the cellular thermal shift assay (CETSA) and photo-affinity labeled chemical proteomics [48,49,50]. Therefore, proteomics can reveal differentially accumulated proteins and their modification patterns associated with signaling metabolite biosynthesis, regulation and functions, aiding in disentangling the relevant complex biological events within cells [51]. One- or two-dimensional gel electrophoresis/mass spectrometry (MS) and liquid chromatography–MS (LC–MS) have been used for the quantification and identification of proteins and potential post-translational modifications [52,53,54]. For instance, by using two-dimensional gel electrophoresis, Decker et al. constructed a two-dimensional protein map of two main fractions of the latex including the cytosolic serum and the sedimented fraction containing the alkaloid-accumulating vesicles isolated from *Papaver somniferum*. Codeinone reductase, an enzyme involved in morphine biosynthesis, within the cytosolic serum fraction was detected following the analysis of the 75 protein spots by internal peptide microsequencing and database matching [55]. Proteins annotated as tocopherol cyclase and prenyltransferases potentially involved in the biosynthesis of orsellinic acid in *Peperomia obtusifolia* could also be identified from the soluble proteins of the different plant tissues using LCMS–IT–TOF-based comparative proteomics analysis coupled with transcriptomics analysis [56]. Furthermore, the recent success in discovering FAD-dependent enzyme-catalyzed intramolecular [4 + 2] cycloaddition in the biosynthesis of natural plant products using chemical probe-based proteomics analysis showcases the utility and applicability of chemical proteomics in secondary metabolite research [57]. Targeted proteomics can also help reveal the rate-limiting steps in certain biosynthetic pathways [58]. At present, the discovery of structures and functions of secondary metabolites using proteomics is often not the first choice due to its relatively higher cost compared to RNA-seq and yet to be established methodologies in studying secondary metabolism. 

#### 2.1.5. Metabolomics—The Node of Multi-Omics for Discovering Signaling Metabolites

Metabolome covers all small molecules including primary and secondary metabolites present in an organism or cell. Metabolomics refers to the systematic analysis of the metabolome of a living system using analytical instruments including liquid chromatography–mass spectrometry (LC–MS) [59], gas chromatography–mass spectrometry (GC–MS) [60] and nuclear magnetic resonance (NMR) [61]. Mass spectrometry (MS)-based metabolomics is the most prevalent method as it can acquire sufficient structural information for compound identification, whilst offering great sensitivity, resolution and compound coverage. The detection of all plant metabolites using one or two methods is impossible due to the enormously diverse chemical and physical properties of plant metabolites. Metabolomics analysis will have to be tailored properly to enable the detection of sufficient compounds for comparative analysis. A few methodological guides have recently been released to aid in MS-based metabolomics analysis [62]. Targeted metabolomics approaches identify and quantify a specific subset of predefined small molecules whilst untargeted metabolomics analysis can collect signals of metabolites (including known and unknown metabolites) that could be detected by detectors for systematic analysis. Comparative metabolomics analysis across different samples allows for the detection of differentially accumulated metabolites, yielding insights into biosynthetic and catabolic dynamics of certain small molecules or pathways. Metabolomics provides direct information regarding the status of metabolites and, thus, serves as a core node for connecting with other omics technologies in discovering plant signaling molecules. It is an essential tool for discovering previously unknown signaling metabolites, especially when starting with plant phenotypes that could possibly arise from metabolites.

#### 2.1.6. Microbiomics—Uncovering Metabolite and Microbe Interactions

Recent studies have proven that the root microbiome, modulated by plant signal metabolites like coumarins, flavones and benzoxazinoids, improves plant stress resilience [63]. Microbiomics investigates all the microorganisms of a given community under various conditions. The main approaches for studying microbial composition are 16S ribosomal RNA (16S rRNA) gene sequencing and shotgun metagenomics sequencing. The bacterial 16S rRNA gene sequences contain species-specific hypervariable regions, which can be amplified, sequenced and then clustered into operational taxonomic units (OUT) for the identification, classification and quantitation of microbes. 16S rRNA amplicon sequencing uses primers for a relatively short genomic region (e.g., V5–V7 zone); therefore, sequencing results can often be annotated to bacterial taxa of relatively higher taxonomic rank. Another microbial community profiling method is next-generation sequencing (NGS)-based shotgun metagenomics sequencing. Total DNA in all organisms present in a given complex mixture are sequenced. This technology compensates for the limits of sequencing the restricted amplicon region in 16S rRNA sequencing, expanding the coverage of microbial DNA to be sequenced, thus, capturing the protein-coding DNA fragments for relatively more accurate functional annotations for microbes present in a sample. The features of 16S rRNA amplicon sequencing and NGS-based shotgun metagenomics sequencing analysis were nicely demonstrated in a recently published work reporting the identification of flavones that function in recruiting the beneficial rhizosphere microbe *Oxalobacteraceae*, which aided maize in acquiring nitrogen under nitrogen deprivation [15].

**Figure 2:**
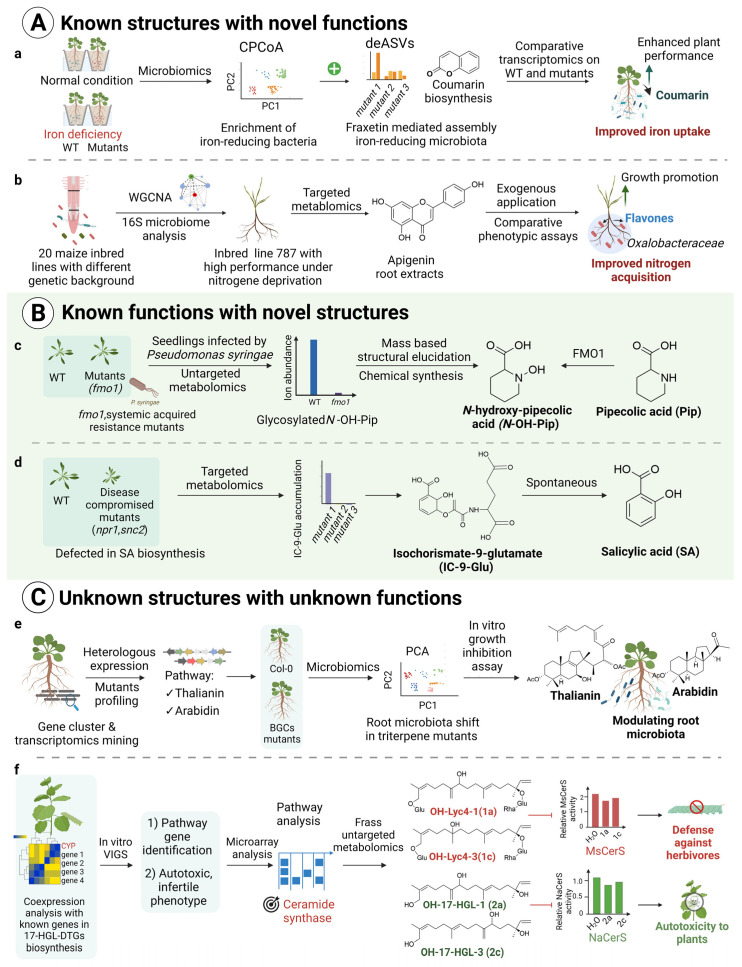
Illustration of the various omics techniques employed in recent discoveries of plant signaling metabolites. The overall experimental designs and key techniques applied for discovering signaling metabolites with different knowledge are depicted in Figure 2. Details for content depicted in (**a**–**f**) can be found in references [75], [15], [76], [77], [78] and [79] respectively.

The different features of each single omics mentioned above can be synergistically. oriented for discovering signaling metabolites, in terms of both structures and functions [64,65,66,67,68,69]. We have seen a surge in plant signaling metabolites being discovered with the aid of multi-omics, particularly in the area of plant–microbe interactions [70,71,72,73,74]. We will illustrate below in more detail how multi-omics techniques were integrated to unveil plant signaling molecules with different levels of knowledge using recent works as examples (Figure 2) to help improve the design of experiments and the application of multi-omics tools in future research.

### 2.2. Multi-Omics-Based Discovery of New Functions of Known Molecules

The integration of multi-omics analysis into studies designed to uncover the metabolic basis of certain phenotypes or traits could lead to the discovery of new functions for some well-known molecules. Coumarins, a family of benzopyrones (1,2-benzopyrones or 2H-1-benzopyran-2-ones), well-known for their defensive role in nature [80], were recently found to act as signaling metabolites in plant–microbe interactions in response to iron deficiency [75,81]. As a well-known class of defense phytochemicals, coumarins protect plants from predation and pathogen infection [80]. The integration analysis of 16S rRNA gene amplicon sequencing along with RNA-seq uncovered that coumarin helps plants to deal with iron limitation by recruiting beneficial soil microbiota. When the culture-independent 16S rRNA gene amplicon sequencing analysis was employed, the impact of coumarin on the root microbiota could be systematically evaluated. Unconstrained principal coordinate analysis (PCoA) of beta diversity constrained (CPCoA) and bacterial community profiles analysis of 16S rRNA sequencing data results indicate that coumarin biosynthesis is important for plant growth and root microbiota assembly in naturally iron-limiting calcareous soil. Moreover, comparative analysis of the amplicon sequence variant (ASV) level in coumarin-deficient mutants with wild type (WT) plants revealed that coumarin fraxetin exerts variable antimicrobial activity on *Burkholderiaceae* strains in iron-limiting soil. Through further root transcriptional profiles and elemental analysis of Col-0 and coumarin-deficient mutant f6′h1 plants under available iron or the unavailable form of FeCl3 media with live or heat-killed synthetic community (SynCom), the role of coumarins, especially fraxetin, in mediating root microbiota for improving plant performance under iron-limiting conditions, was uncovered [75] (Figure 2a). This new function of coumarins would have not been discovered had the integrated analysis of microbiomics and transcriptomics were not applied.

Combined transcriptomics, metabolomics and microbiomics analysis were also employed to discover the hidden roles of other signaling molecules such as flavones [15], benzoxazinoids [82] and strigolactones [73] in mediating plant and microbe interactions. Flavones are phenolic compounds that have functions in plant signaling, defense and adaptation to stress conditions [83]. In a recent study designed to unlock mechanisms underlying beneficial interactions between plants and rhizosphere microorganisms, flavones synthesized in maize roots were found to be capable of recruiting rhizosphere *Oxalobacteraceae* bacteria to improve maize performance under nitrogen deprivation [15]. Hundreds of RNA-seq datasets together with their corresponding rhizosphere microbiome data from three longitudinal zones of the crown roots of 20 inbred lines of maize with significantly different genetic backgrounds were generated. Using WGCNA network analyses on root RNA-seq datasets, phylogenetic and genotype-specific gene modules that contained gene sets with similar expression patterns across all samples were identified. Correlation analysis of the expression module with maize genotypes, phenotypes and microbiome data enabled the authors to target a specific module that displayed the highest correlation with *Oxalobacteraceae* enriched in the root of the high-performance inbred line of maize 787 under nitrogen deprivation. The fact the flavone synthase displayed the highest modular connectivity within this module further suggests that flavones might play a role in mediating the assembly of a beneficial root microbiota for the high-performance inbred line of maize 787. To further confirm whether flavones act as a signaling molecule under nitrogen deprivation, targeted metabolite profiling of maize root extracts of the high- and low-performance maize genotypes, together with comparative phenotypic assays of wild type maize and chalcone synthesis mutants as well as complementation experiments with exogenous flavonoids further identified the roles of root-secreted flavones, especially apigenin, in recruiting *Oxalobacteraceae* bacteria for promoting lateral root development and nitrogen uptake in maize [15] (Figure 2b). The new function of flavones would not have been identified without an in-depth correlation analysis of transcriptomics and microbiome 16S rRNA sequencing data. Therefore, new functions of known plant metabolites could potentially be uncovered from the studies aiming to explore the mechanisms underpinning certain phenomena or traits. 

### 2.3. Multi-Omics-Based Discovery of Unknown Molecules with Known Functions

An untargeted metabolomics approach is essential to uncover novel molecules that might have given rise to certain biological functions. The comparative metabolic profiling of samples with and without biological activities can capture the chemical differences in these samples unbiasedly, enabling the design of experiments to further investigate the structures and biological activities of these chemicals. Many previously unknown molecules have recently been identified using an untargeted metabolomics approach [76,77,84,85]. One notable example is the discovery of *N*-hydroxy-pipecolic acid as a mobile signaling metabolite that induces systemic disease resistance in *Arabidopsis* [76] (Figure 2c). This metabolite was identified via comparative metabolic profiling of the *Arabidopsis* Flavin-Dependent Monooxygenase 1 (FMO1) mutant that is deficient in systemic acquired resistance (SAR) with wild type *Arabidopsis* plants. Although FMO1 has been identified as a key component in mediating the SAR against pathogens for *Arabidopsis* [86], the chemical basis of FMO1 remains elusive, primarily due to the unprecedented nature of the biosynthetic pathway. Untargeted metabolomics analysis nicely revealed a major mass signal present in wild type plants in response to *Pseudomonas syringae* treatment but absent from all *fmo1* mutant plants. Further structural elucidation based on mass spectra fragmentation and synthetic standards confirmed the chemical identity of the mass signal as glycosylated *N*-hydroxy-pipecolic acid, suggesting that FMO could hydroxylate pipecolic acid to form *N*-hydroxy-pipecolic acid, which can be further glycosylated in planta [87]. Having mutant plants of genes involved in certain biological events would be very helpful for uncovering the chemical basis contributing to the biological activity of the gene under investigation. The discovery of isochorismate-9-glutamate as an important intermediary in the biosynthesis of salicylic acid exemplifies this strategy [77]. The disease compromised *Arabidopsis* mutant *npr1* (nonexpressor of pathogenesis-related genes, NPR1) with reduced salicylic acid content and the snc2 (suppressor of npr1-1, constitutive 2) mutant which displays an autoimmune phenotype with an excess of salicylic acid were used to perform comparative untargeted metabolomics analysis, which successfully identified new intermediaries for salicylic acid biosynthesis [77] (Figure 2d). MS-based untargeted metabolomics analysis provides ample structural information regarding the chemical signals being detected, enabling annotation of the metabolites with different levels of confidence, though it is still challenging to annotate most of the chemical signatures detected by untargeted MS [88]. Synthetic standards or the NMR spectra of purified chemicals are normally required to confirm the identity of unknown compounds. Nevertheless, advances in plant metabolomics, both technical and computational, will greatly facilitate the identification and delineation of chemical signals underlying gene functions [89], leading to the discovery of novel compounds that contribute to certain biological functions.

### 2.4. Multi-Omics-Based Discovery of Unknown Molecules with Unknown Functions

Discovering novel molecules with defined biological activities has been an ongoing task in natural product research. The advent and development of multi-omics technology, especially genomics, have revolutionized the way unknown natural products are discovered [17,90], shifting from phytochemistry-based isolation and functional evaluation to genome- and transcriptome-based structural and functional mining. Genomic and transcriptomic features underlying the biosynthesis of plant natural products can enable the fast discovery of previously unknown plant metabolites when coupled with efficient heterologous expression systems. Alternatively, function oriented/guided studies of genes predicted to be involved in metabolite biosynthesis, regulation or transport can often unearth unknown metabolites with novel functions. Notable examples include the recent discovery of a previously unknown specialized triterpene biosynthetic network involved in selectively modulating *Arabidopsis* root microbiota [78], a new cyanogenic metabolite in *Arabidopsis* required for inducible pathogen defense [84] and hydroxylated diterpenoids involved in plant defense [79].

Gene clustering is increasingly demonstrated to be an important genomic feature that can be utilized for the facile discovery of plant signaling metabolites [91]. Plant biosynthetic gene clusters provide a great entry point to discover and elucidate previously unknown biosynthetic pathways as multiple biosynthetic genes functioning in the same pathway can be easily identified at the same time. The specialized triterpene biosynthetic network operating in Arabidopsis roots was recently discovered using this approach, starting with the heterologous functional characterization and untargeted metabolomics analysis of root-expressed triterpene biosynthetic cluster genes and their mutants to uncover novel triterpene chemical structures. This was followed by 16S rRNA microbiomics analysis of triterpene-deficient mutants and wild type *Arabidopsis* root microbial communities to delineate the function of the triterpene biosynthetic network in modulating *Arabidopsis* root microbiota [78] (Figure 2e). Transcriptomics data enabled the discovery of other co-expressed biosynthetic genes that are not clustered with the core cluster genes but function in the same thalianin and arabidin biosynthetic pathways. This provided hints on the functions of the triterpene biosynthetic network, leading to further microbiomics analysis [78]. 

Similar to genomic features, transcriptomic features underlying plant metabolite biosynthesis can serve as an entry point to probe unknown plant metabolisms. The fact that the synthesis of plant signaling metabolites is responsive to external stimuli allows investigation of the transcriptomic alteration of biosynthetic genes coding for the synthesis of cryptic metabolites. Using transcriptomics data as the entry point for discovering plant signaling metabolites offers many advantages: (i) relative low cost of RNA-seq sequencing and ease of transcriptome assembly as compared to genome sequencing and assembly; (ii) amplifiable signals of transcriptomic changes can be captured with high accuracy and relatively low amounts of plant materials in contrast to the relatively large quantity required for untargeted metabolomics analysis; (iii) functional annotation of transcriptomics sequences with higher prediction accuracy in comparison to untargeted metabolomics analysis. The new cyanogenic metabolite in *Arabidopsis* required for inducible pathogen defense was discovered based on the untargeted metabolomics analysis of mutants of genes involved in defense against pathogens as identified from the pathogen-induced transcriptomics data analysis [84].

It is clear that untargeted metabolomics analysis has to be carried out to correlate with transcriptomics data for the identification of structurally unknown compounds. In some cases, untargeted metabolomics analysis on the organism (e.g., insects) interacting with plants can also provide cues leading to the identification of unknown metabolites with novel functions [79,89]. In a recent study aiming to uncover the metabolic basis for defense and autotoxicity of 17-hydroxygeranyllinalool diterpene glycosides (17-HGL-DTGs), the authors identified the ceramide synthase inhibition activity of modified diterpene glycosides via untargeted metabolomics analysis of the insect *Manduca sexta* fed with tobacco plants containing normal and compromised diterpene glycoside levels as well as its frass [79,89]. *Manduca sexta* fed with tobacco plants containing normal diterpene glycoside levels accumulated significantly more long chain bases, which are substrates of ceramide synthase inhibited by the diterpene glycosides, than those fed with compromised levels of the diterpene glycosides. Moreover, the frass of *M. sexta* fed with tobacco containing the normal level of diterpene glycosides also accumulated more modified 17-HGL-DTGs than those fed with tobacco containing compromised levels of diterpene glycosides. The identification of modified 17-HGL-DTGs as novel compounds and their activities in inhibiting ceramide synthase led to the further discovery of the toxicity of modified diterpenes (i.e., hydroxylated hydroxygeranyllinalool diterpenes) on tobacco plants. It is interesting to note that the ceramide synthase inhibition activity of 17-HGL-DTGs on *M. sexta* was identified based on cues obtained from comparative transcriptomics analysis of wild type tobacco and autotoxic tobacco mutant plants [79] (Figure 2f). Therefore, an untargeted metabolomics approach is indispensable for the identification of unknown compounds, and when coupled with transcriptomics analysis can often unearth unknown compounds with novel biological activities.

## 3. Breaking the Limitation of Multi-Omics: Future Perspective for Accelerated Discovery of Plant Signaling Molecules

Multi-omics technology has greatly facilitated the discovery of plant signaling metabolites in many aspects; however, technical limitations in individual omics techniques still pose challenges to their application. With regard to genomics, although long-read sequencing such as PacBio [92] and Nanopore [93] sequencing technologies have improved read length and, therefore, genome assembly, to some extent, the high levels of heterozygosity, complex polyploidy and the unusually high repeat content of plant genomes are still challenges impeding accurate genome assembly and annotation [94,95]. An increasing number of plant genomes have been sequenced, yet a reasonable number of which were poorly assembled and annotated (both structurally and functionally) or with low sequence quality. Functional genomics relies heavily on the sequence information of a genome; assembly errors create hurdles for the functional prediction of biosynthetic genes or gene clusters, leading to incorrect identification of plant biosynthetic gene clusters for functional validation using currently available bioinformatics tools. For instance, the discovery of plausible functional biosynthetic gene clusters would be undermined if the assembly is only at the scaffold level rather than chromosome level as the biosynthetic genes potentially forming a gene cluster might span across multiple scaffolds. Moreover, incorrect sequence information can also result in cloning issues due to not being able to design appropriate primers as a result of missing or incorrect sequence information of a gene in a plant genome. Therefore, further technical development is desired to improve read length and the accuracy of genome sequencing techniques. 

Similarly, sequencing read length and accuracy also affect de novo transcriptome assembly, functional annotation, gene cloning and functional validation, especially for those plant species without a sequenced genome. Currently, RNA-seq data are primarily generated using second-generation Illumina sequencing due to the low cost and relatively well-developed analysis pipeline [96]. Single-molecule Nanopore RNA and PacBio sequencing can significantly improve read length [97], yet the cost is still relatively high in comparison to Illumina sequencing. These problems are expected to be resolved in the near future with the development of long read sequencing technologies and continuously deceasing sequencing cost. Another limitation associated with transcriptomics mining for signaling molecule discovery is the resolution of data. RNA-seq data were previously generated from the bulk RNA of plant tissues, which inevitably include much transcript noise from cells where the gene of interest is not expressed [98]. The development of the single-cell sequencing technique has enabled RNA sequencing at single-cell or cell-type levels, removing undesired transcript noise from unwanted cells, thereby yielding the much finer resolution of data for dissecting gene functions in specific cells and allowing better correlations of gene functions using co-expression analysis [99]. This will be particularly useful for dissecting the functions of known/unknown metabolites as well as uncovering their biosynthesis.

Although single-cell RNA sequencing techniques have greatly expanded multi-omics application, yielding hidden and more complete mechanistic insights, the development of single-cell metabolomics, in comparison, still lags far behind. This is primarily due to the fact that metabolite signals could not be amplified the same way as DNA and RNA and instrument sensitivity is not yet up to the point of detecting comprehensive sets of metabolites within a single cell [100]. MS-based metabolomics is the most prevalent metabolomics approach, yet detection of metabolites with current instrument settings including ionization methods still face many challenges, although sophisticated sensitive instruments such as Orbitrap and time-of-flight (ToF) mass spectrometers have been widely applied. Besides sensitivity issues, the annotation of the metabolite signals from MS-based metabolomics data also represents a significant problem for metabolomics analysis [59]. Currently, the compound identity of MS-based metabolomics is assigned primarily based on accurate mass and MS/MS fragmentation data available from various databases, including METLIN [101], PubChem [102] and mzCloud. The confidence level and accuracy for such annotation are still relatively low, especially for the numerous unknown metabolites present in a plant matrix. The annotation issue is expected to be alleviated with the expansion of characterized chemical entities in the databases, standardization of instrumentation parameters and newly developed artificial intelligence including machine learning algorithms that can also be incorporated to aid in the annotation of metabolites based on mass features, especially MS/MS fragmentation patterns from metabolomics experiments [103]. The power of extracting features from metabolomics data is already evident from the development of the molecular networking approach, which clusters mass fragments with different degrees of similarities to facilitate the annotation of mass spectrum signals and has already found applications in many areas [86,104]. Integrating patterns and features from different levels of omics for machine learning may generate models that can streamline the process of multi-omics analysis and speed up the process of the discovery of plant signaling molecules [105].

It is foreseeable that the discovery of plant signaling molecules will accelerate in the near future with the increasing availability of omics tools. Novel entities and functions of plant signaling molecules at single-cell or cell-type levels will be an important research direction going forward. In addition, the discovery of plant signaling molecules involved in the interaction between plants and environments or other living organisms will also be a trend in the field with future research. With a better understanding of the functions of plant signaling molecules, their utility will be further exploited, increasing the potential of commercialization, especially in agriculture-related areas. This will also fuel the development of sustainable production technologies including synthetic biology.
